# Nutritional status of Tajik children and women: Transition towards a double burden of malnutrition

**DOI:** 10.1111/mcn.12886

**Published:** 2019-11-08

**Authors:** Tanja Barth‐Jaeggi, Lizelle Zandberg, Mutribjon Bahruddinov, Sabine Kiefer, Sherali Rahmarulloev, Kaspar Wyss

**Affiliations:** ^1^ Swiss Tropical and Public Health Institute Basel Switzerland; ^2^ University of Basel Basel Switzerland; ^3^ Centre of Excellence for Nutrition North‐West University Potchefstroom South Africa; ^4^ UNICEF Tajikistan Dushanbe Tajikistan; ^5^ Ministry of Health and Social Protection (MoHSP) Tajikistan Dushanbe Tajikistan

**Keywords:** children 0–5 years, double burden of malnutrition, malnutrition, micronutrient deficiency, nutritional status, overweight, Tajikistan, women of childbearing age

## Abstract

The double burden of malnutrition, an emerging concern in developing countries, can exist at various levels: individual, household, and population. Here, we explore the nutritional status of Tajik women (15–49 years) and children (5–59 months) focusing on overweight/obesity along with undernutrition (underweight, stunting, and micronutrient deficiencies). For this, nutritional markers (haemoglobin (Hb), transferrin receptor (TfR), serum ferritin (Sf), retinol binding protein (RBP), vitamin D, serum folate, and urinary iodine), height, and weight were assessed from 2,145 women and 2,149 children. Dietary intake, weaning, and breastfeeding habits were recorded using a 24‐hr recall and a questionnaire. Overweight (24.5%) and obesity (13.0%) are increasing among Tajik women compared with previous national surveys (2003 and 2009). Prevalence of iron deficiency and anaemia was 38.0% and 25.8%, respectively; 64.5% of women were iodine deficient, 46.5% vitamin A deficient, and 20.5% had insufficient folate levels. Women in rural areas had significantly lower iron status and body mass index and higher iodine intake compared with urban areas; 20.9% of children were stunted, 2.8% wasted, 6.2% underweight, 52.4% iron deficient, and 25.8% anaemic; all more prominent in rural areas. Dietary diversity was higher among urban women. Intraindividual or household double burden was not seen. In summary, double burden of malnutrition constituted an increase in overweight among women, especially in urban areas, and persisting levels of undernutrition (stunting, iron, and vitamin A deficiency), predominately in rural areas. A holistic, innovative approach is needed to improve infant and young children feeding and advise mothers to maintain an adequate diet.

## Abstract 2

Rituximab was digested with immobilized pepsin and papain to yield F(ab')2 and Fab' fragments respectively. Fragments were conjugated with p‐SCN‐Bn‐NOTA and immunoconjugates labeled with Ga‐68. Specificity of radioimmunoconjugates was ascertained by cell binding and inhibition assays with unlabeled antibody.

**Key messages**

- This national survey reports the nutritional status of Tajik women of childbearing age and children under 5 years.
- There is a high prevalence of micronutrient deficiencies (iron, iodine, and vitamin A) among the study population.
- Over the last decade and along persistent undernutrition in children, a marked increase in overweight and obesity in women is observed leading to a double burden of malnutrition in the Tajik population.
- The double burden of malnutrition is mainly represented by increasing overweight and obesity in women and ongoing undernutrition in children.
- The rural population is more affected by undernutrition and the urban population by the increase of overweight.
- A multidisciplinary collaborative approach is needed to tackle the public health problem of malnutrition in the Tajik maternal and child population.

## 1 INTRODUCTION

Undernutrition derives from insufficient energy and nutrients intake to meet an individual's needs and is often associated with underweight, stunted, wasted, or suffering from micronutrient deficiencies (Maleta, 2006; World Health Organization [WHO], 2018b). Undernutrition is a widespread health problem, particularly affecting the low‐ and middle‐income countries. Adequate maternal nutrition during pregnancy and lactation is important for the promotion of optimal health during early development of the infant. Stunting, as a result of chronic malnutrition, can only be fully reversed if addressed efficiently within the first 2 years of life (Black et al., 2008). Iron deficiency (ID) and the consequential ID anaemia (IDA) in early life can lead to poor cognitive and motor development (Sachdev, Gera, & Nestel, 2005). Iodine deficiency leads to insufficient production of thyroid hormone, which may result in foetal brain damage and impairs cognition and growth in early life (Zimmermann & Boelaert, 2015), mental function, reduced work productivity, and toxic nodular goitre in adults (Taylor et al., 2018; Zimmermann & Boelaert, 2015). Folic acid plays a major role in foetal neural tube formation (van Gool, Hirche, Lax, & De Schaepdrijver, 2018). Emerging evidence on nutrition during pregnancy and early development suggests undernutrition in utero and in early life may predispose an individual to be overweight and susceptible to the development of noncommunicable diseases (diabetes and heart disease) later in adult life (WHO, 2018a).

Paradoxically, many parts of the world are currently exposed to an overweight and obesity epidemic, with over 39% of adults being overweight worldwide, caused by an energy imbalance between consumption and expenditure (WHO, 2018c). As a consequence, noncommunicable diseases like cardiovascular diseases, diabetes, musculoskeletal disorder, and some cancers are increasing (Collins et al., 2018; Igel, Saunders, & Fins, 2018; Steele et al., 2017). This fast increase in overweight and obesity along with a slow decrease or stagnancy in undernutrition (wasting, stunting, and micronutrient deficiencies) experienced in many low‐ and middle‐income countries (Freire, Silva‐Jaramillo, Ramirez‐Luzuriaga, Belmont, & Waters, 2014; Haddad, Cameron, & Barnett, 2015; WHO, 2018a) leads to the so‐called double burden of malnutrition (Kosaka & Umezaki, 2017). It can exist at various levels: At individual level, for example, as overweight along with nutritional deficiencies; at household level, with underweight or stunted children and overweight adults; or at population level, with the coexistence of undernutrition and overweight.

Previous Tajik National Nutritional Surveys (TNNS) have been conducted in 2003 and 2009 (Ministry of Health Republic of Tajikistan and United Nations Children's Fund [UNICEF], 2010; UNICEF, 2003). In 2009, an association between overweight/obesity and anaemia in children under 5 years was investigated, but no association could be confirmed, and with this, no intraindividual double burden of malnutrition (Crivelli et al., 2018). Further, large surveys were the 2005 Multiple Indicator Cluster Survey (MICS) and the 2012 and 2017 Demographic and Health Surveys (DHS), which offered a time trend on nutrition status based on anthropometrics (State Committee on Statistics of the Republic of the Tajikistan United Nations Children's Fund (UNICEF), 2005; Statistical Agency under the President of the Republic of Tajikistan et al., 2013; 2018). In the 2016 TNNS, a large panel of nutritional indicators was assessed together with anthropometrics, offering the opportunity for a solid analysis of malnutrition in women and children. Here, we explore the nutritional status of children under 5 years and women of childbearing age and patterns of the double burden of malnutrition that Tajikistan is facing.

## 2 MATERIALS AND METHODS

### 2.1 Study population and design

This survey was based on a national cross‐sectional cluster sample design using population estimates from the 2010 census extrapolated to 2016. Sampling was conducted in a two‐stage approach to obtain representative data for Tajikistan (stratified by urban and rural areas) and among the four administrative regions (oblasts) of the country (Districts of Republican Subordination [DRS], Sughd, Khatlon, Gorno‐Badakhshan [GBAO]) and Dushanbe (DRS).

The sampling estimates and procedures derive from the 2009 TNNS (Ministry of Health Republic of Tajikistan and UNICEF, 2010), using the formula: *c* = (((*t* ^2^·*p* (1 − *p*))/*m* ^2^)·*d*)/*nh* + 10%, where *c* = required number of cluster, *t* = confidence level at 95% (standard value of 1.96), *p* = estimated 50% prevalence of micronutrient deficiencies, *m* = margin of error at 6.5% , *d* = design effect of 1.75, and *nh* = number of household by cluster. Therefore, the necessary sample size was estimated to be 2,160 children and 2,160 women including anticipated 10% dropouts or sample loss (Tajikistan, 2010).

A total of 36 clusters per oblast were selected with the probability of selection within strata being proportional to size and clusters consisting of urban and rural domains proportional to their distribution in the respective oblast. In every selected cluster (village and community), 12 children between 6 and 59 months and 12 nonpregnant women of childbearing age (15–49 years) were randomly selected. This selection relied on two mechanisms, depending if the cluster was involved in the World Bank's “Poverty Diagnostics of Water Supply, Sanitation and Hygiene Condition in Tajikistan” survey (World Bank, 2017). If this was the case, the same households were visited, and if needed, a random walk was done to achieve the envisaged sample size. If the respective cluster was not part of the WB survey, a local census list was used to randomly select 20 households (UNICEF, 2016).

The survey instruments, based on the questionnaires used in 2009, were developed by UNICEF and Swiss Tropical and Public Health Institute to assure consistency with previous surveys. These instruments were updated to provide the most relevant information on the health and nutritional status for children under 59 months and women of reproductive age. For the assessment of dietary consumption patterns within the past 24 hr, the approach and methodology used were as described in “Minimum Dietary Diversity for Women—A Guide to Measurement” (FAO and FHI 360, 2016). The minimum dietary diversity (MDD) indicator is based on the following food groups: (a) grains, white roots and tubers, and plantains; (b) pulses (beans, peas, and lentils); (c) nuts and seeds; (d) dairy; (e) meat, poultry, and fish; (f) eggs; (g) dark green leafy vegetables; (h) other vitamin A‐rich fruits and vegetables; (i) other vegetables; (j) fruits (FAO and FHI 360, 2016). The questionnaires were translated into Tajik and back‐translated into English to check for any misinterpretations.

### 2.2 Data collection

In November 2016, 12 teams (each including a clinician, an interviewer for the face‐to‐face interviews, a laboratory technician in charge of the biological samples, and a driver) collected data, blood, and urine samples. Data were collected using electronic tablets and the Open Data Kit software (https://opendatakit.org/).

The face‐to‐face interviews covered the topics on household characteristics, food security, child health, infants feeding, and weaning practices, as well as dietary habits/intakes of the women using a 24‐hr recall questionnaire. Further, household salt was tested for its iodine content using the MBI rapid test kit (UNICEF supply cat nr: S0008193).

The weight (electronic scale mother/child, 150 kg × 100 g; UNICEF supply cat nr: S0141021) and height/length (portable measuring board; UNICEF supply cat nr: S0114530) of women and children were recorded. Capillary blood (approximately 400 μl) was collected using serum separating gel tubes (Microvette®, Sarstedt) and stored in a cooling box until centrifugation at the nearest district or oblast hospital the same day. The serum was transferred to light‐protected tubes (Microtainer™, Sarstedt) and frozen at −20°C until analysis. Hb was measured on spot using a HemoCue device (HemoCue AB, Ängelholm, Sweden). The clinicians provided clinical feedback regarding the anaemia status (Hb value), and anaemic women and children were advised to attend nearby health facilities.

For the urinary iodine concentration (UIC) analysis, containers were distributed to the women or the guardians for urine collection. Urine sampling from children (6–59 months) was done, whenever possible, from the traditional cradle (Gavora, where the urine is collected in a pot under the cradle). The urine samples were transferred to a labelled 20‐ml falcon tubes and also frozen at −20°C until analysis.

### 2.3 Biochemical analyses

The laboratory analyses were carried out by the Research Laboratory of Preventive Medicine of the Republic of Tajikistan in Dushanbe in early 2017. Serum samples were analysed using enzyme‐linked immunosorbent assays to assess SF (BioVendor GmbH, cat nr: RCD012R), TfR (BioVendor GmbH, cat nr: RD194011100), C‐reactive protein (CRP, BioVendor GmbH, cat nr: RAP001), RBP (Abcam, cat nr: ab196264), vitamin D (Enzo Life Sciences, Inc., cat nr: ADI‐900‐215), and serum folate (antibodies‐online Inc., cat nr: ABIN857086). Analyses were conducted according to the manufacturer recommendations (including high and low controls on each plate). In addition, an internal control, a pooled serum sample, was included with each batch and used for interplate variation analysis and quality control assessments.

UIC in spot urine was measured using the Pino modification of the Sandell–Kolthoff reaction with spectrophotometric detection (Caldwell, Makhmudov, Jones, & Hollowell, 2005; Pino, Fang, & Braverman, 1996). All analyses were done using nanopure grade water, and all laboratory glassware and plasticware were acid washed before use. Internal controls were used for the UIC analysis.

### 2.4 Statistical analysis

Z‐scores were calculated with the World Health Organization (WHO) reference for children aged 0–5 years, using the WHO Anthro software v3.2.2. Stunting was defined as a low length/height‐for‐age z‐score (<−2 for moderate or <−3 for severe), wasting as a low weight‐for‐height z‐score (<−2 for moderate or <−3 for severe), and underweight as low weight‐for‐age z‐score (<−2 for moderate or <−3 for severe). The body mass index (BMI) was calculated using weight and height: BMI = kg/m^2^. Anthropometrical data of previous large surveys (TNNS 2003 and 2009; MICS 2005; DHS 2012, 2017) were used to show time trends.

To adjust for altitude differences, Hb measurements were corrected according to the formula suggested by Sullivan et al.: Hb = −0.032·(altitude·0.0032808) + 0.022·(altitude·0.0032808)^2^ (Sullivan, Mei, Grummer‐Strawn, & Parvanta, 2008). Inflammation sensitive markers were adjusted using the Thurnham correction factors of 0.77 for SF (Thurnham et al., 2010) and 1.14 for RBP (Thurnham, McCabe, Northrop‐Clewes, & Nestel, 2003) when there was a concurrent elevated CRP (>5 μg/ml). These cut‐offs were applied to determine nutritional status and inflammation in women (a) and children (b): anaemia, (a: Hb < 12 g/dl and b: <11 g/dl (WHO, 2011a)), ID (a: SF < 15 ng/ml or TfR > 3.3 μg/ml and b: SF < 12 ng/ml or TfR > 3.3 μg/ml (WHO/CDC, 2007)), IDA (a: Hb < 12 g/dl plus SF < 15 ng/ml or TfR > 3.3 μg/ml and b: Hb < 11 g/dl plus SF < 12 ng/ml or TfR > 3.3 μg/ml (WHO/CDC, 2007)), iodine deficiency (a and b: UIC < 100 μg/L (WHO, 2013)), vitamin A deficiency (a and b: RBP ≤ 0.70 μmol/L (WHO, 2011b)), vitamin D deficiency (b: vitamin D < 19.6 ng/ml (Basatemur, Horsfall, Marston, Rait, & Sutcliffe, 2017; Braegger et al., 2013) folate insufficiency (a: Folate<3 ng/ml (WHO, 2015)) and inflammation (a and b: CRP > 5 μg/ml (CRP, BioVendor, RAP001 manual); Tables 1 and 2). Body iron stores were calculated from the ratio of TfR to SF according to the following equation by Cook et al.: body iron (mg/kg) = −[log_10_ (TfR·1000/SF) − 2.8229)]/0.1207 (Cook, Flowers, & Skikne, 2003).

**Table 1:** Prevalence of micronutrient deficiencies among women (15–49 years) by region and nationally

	Anaemia, %	ID, %	IDA, %	Iodine deficiency, %	Vitamin A deficiency, %	Folate insufficiency, %	Inflammation, %
Dushanbe	20.0	32.2	10.1	72.8	38.5	15.6	88.9
Khatlon	34.4	30.3	15.2	51.4	66.6	22.8	80.8
Sughd	19.4	45.4	11.2	43.2	44.4	14.6	79.1
DRS	22.2	41.2	14.8	65.0	36.4	25.7	76.9
GBAO	31.8	40.4	17.9	74.1	47.7	35.3	86.8
rural	27.8	40.2	15.3	58.7	47.7	24.9	80.5
urban	21.2	34.0	10.9	66.8	44.3	18.8	86.1
National (weighted)	25.8	38.0	13.8	61.5	46.5	20.5	82.5
Indicator and cut‐off	Hb < 12 g/dl (WHO, 2011a)	SF < 15 ng/ml or TfR > 3.3 μg/ml (WHO/CDC, 2007)	Hb < 12 g/dl plus SF < 15 ng/ml or TfR > 3.3 μg/ml (WHO/CDC, 2007)	UIC < 100 μg/L (WHO, 2013)	RBP ≤ 0.70 μmol/L (WHO, 2011b)	Folate<3 ng/ml (WHO, 2015)	CRP > 5 μg/ml

*Note*. Hb adjusted to altitude (Sullivan et al., 2008); SF adjusted for inflammation (Thurnham et al., 2010); RBP adjusted for inflammation (Thurnham et al., 2003).

Abbreviations: CRP, C‐reactive protein; Hb, haemoglobin; ID, iron deficiency; IDA, iron deficiency anaemia;SF, serum ferritin; TfR, transferrin receptor; UIC, urinary iodine concentration.

**Table 2:** Prevalence of micronutrient deficiencies among children (6–59 months) by region and nationally

	Anaemia, %	ID, %	IDA, %	Iodine deficiency, %	Vitamin A deficiency, %	Vitamin D deficiency, %	Inflammation, %
Dushanbe	16.5	44.0	9.5	62.2	21.0	19.4	67.6
Khatlon	29.4	54.5	18.6	48.9	37.0	22.5	59.5
Sughd	23.4	67.2	17.0	42.6	49.7	8.6	64.0
DRS	25.5	38.4	11.9	57.2	41.5	9.3	63.7
GBAO	43.4	59.0	28.7	67.7	36.0	2.6	65.4
rural	31.7	55.1	20.1	55.3	46.4	9.6	63.8
urban	19.4	47.3	11.0	57.1	29.3	17.9	64.5
National (weighted)	25.8	52.4	16.9	50.9	37.0	12.4	64.0
Indicator and cut‐off	Hb < 11 g/dl (WHO, 2011a)	SF < 12 ng/ml or TfR > 3.3 μg/ml (WHO/CDC, 2007)	Hb < 11 g/dl plus SF < 12 ng/ml or TfR > 3.3 μg/ml (WHO/CDC, 2007)	UIC < 100 μg/L (WHO, 2013)	RBP ≤ 0.70 μmol/L (WHO, 2011b)	Vitamin D < 19.6 ng/ml (Basatemur et al., 2017; Braegger et al., 2013)	CRP > 5 μg/ml

*Note*. Hb adjusted to altitude (Sullivan et al., 2008); SF adjusted for inflammation (Thurnham et al., 2010); RBP adjusted for inflammation (Thurnham et al., 2003).

Abbreviations: CRP, C‐reactive protein; Hb, haemoglobin; ID, iron deficiency; IDA, iron deficiency anaemia;SF, serum ferritin; TfR, transferrin receptor; UIC, urinary iodine concentration.

Statistical analyses were done in Stata version 14.0. Emphasis was put on investigating national, household, and intraindividual associations and differences of malnutrition indicators, with a special focus on overweight and undernutrition (underweight, stunting, wasting, and micronutrient deficiencies). Differences in prevalence were assessed using Pearson's chi‐square tests and differences of absolute variables using *t* tests. To display nationally representative data, the demographic weight of the different oblast in their rural and urban areas was taken into account, and national data weighted accordingly. To assess double burden at household level, regressions were conducted looking into correlations of BMI of women and anthropometrical or nutritional markers of children of the same household (generalised least squares random effect model).

## ETHICS

Ethical clearance was received by the Ministry of Health and Social Protection of the Republic of Tajikistan (MoHSP, N# 804, approved 24.10.2016) and Ethics Committee of Northwestern and Central Switzerland (EKNZ, 2016‐00543, approved 28.10.2016). All interviewees gave written informed consent before the interview and collection of biological samples. Legal guardians gave informed consent on behalf of the children. No treatment or incentive was given to the study participants.

## 3 RESULTS

**Figure 1:**
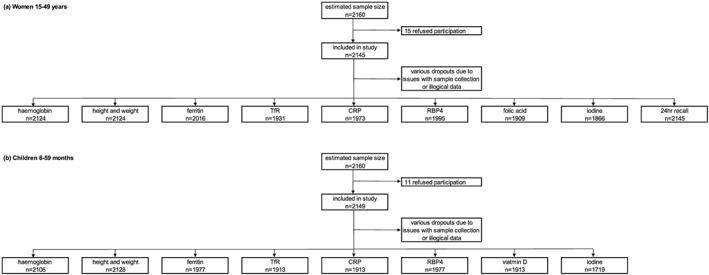
Study participants flow of estimated sample size, totally included participants, and number of indicators analysed of (a) women (15–49 years) and (b) children (6–59 months)

A total of 2,149 children aged 6–59 months and 2,145 nonpregnant women of childbearing age (15–49 years) were included in this study (Figure 1). In addition, feeding practices of 232 infants aged 0–6 months were recorded. The study sample represented mostly individuals living in rural areas with the exception of Dushanbe, which included only urban dwellings. Overall, 17.3% of household heads were female, and the majority had secondary education (10 and 11 grades). Remittances are an essential source of income in Tajikistan with 31% of households, indicating it as their main source of cash income, followed by the official salary (21.4%), farming/livestock (19.3%), and private business (12.8%). Few respondents had no cash income (6.9%) or were living from pension/social aid (4.0%). Evidently, two out of three households were growing food crops (63.7%) or kept livestock (62.3%) for complementing their own consumption. Except in Dushanbe, where only 1.3% of the respondents reported to grow their own food, and 1.0% kept livestock.

### 3.1 Dietary intake and nutritional habits

**Figure 2:**
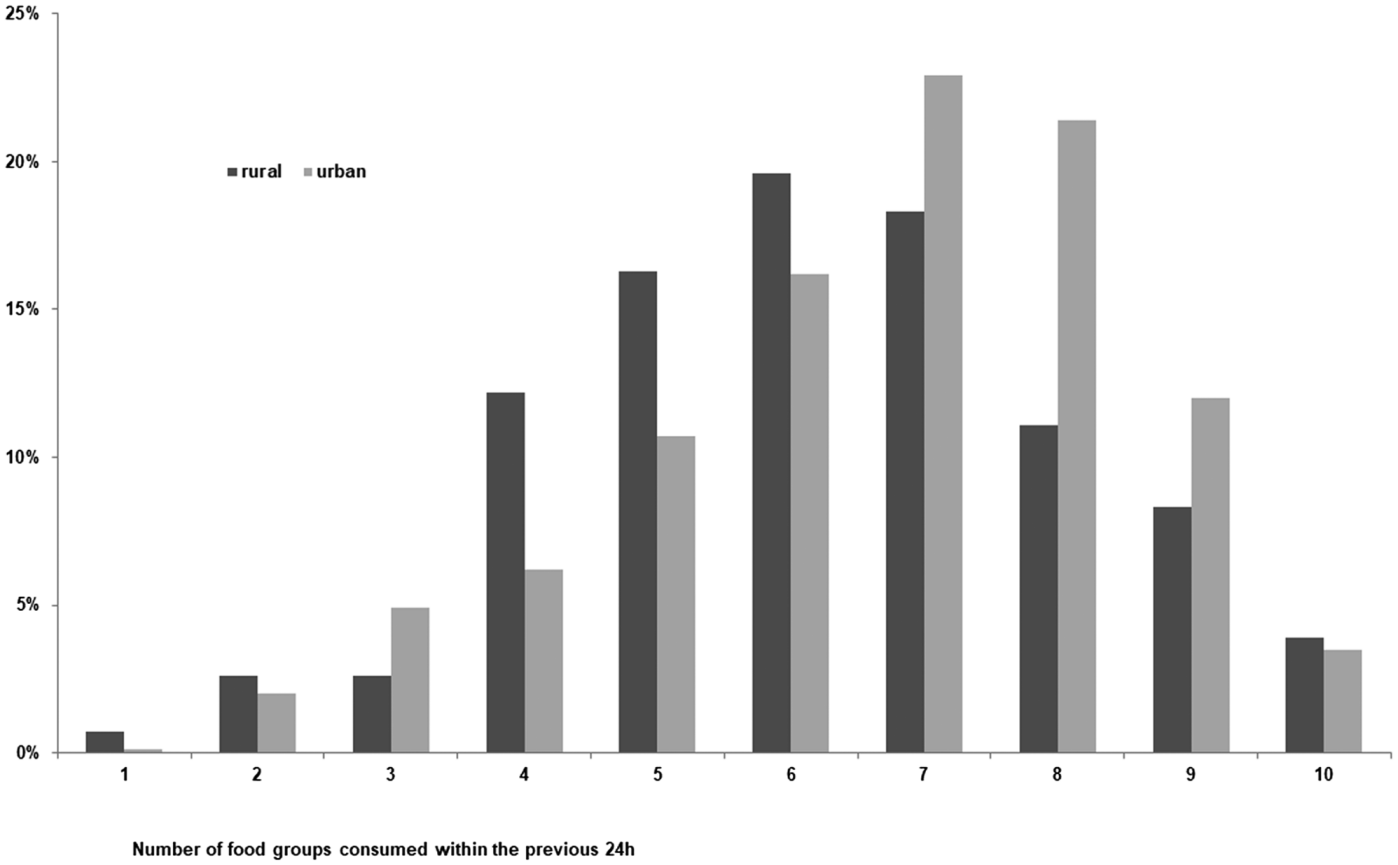
Dietary diversity scores of women 15–49 years (out of 10 food groups)

The diet of Tajik women of childbearing age was rich in cereals (wheat, bread, rice, pasta, and biscuits), potatoes, and other roots or tubers, as well as fats and oil, vegetables, and sweets, which were consumed by over 80% of the women in the previous 24 hr. Further, over 60% reported to have eaten dairy products and meat, but only 3.9% consumed fish. Most women reported drinking mainly plain water and tea or coffee. At the national level, 80.7% of women consumed a minimum of five food groups as defined by the MDD score in the previous 24 hr (FAO and FHI 360, 2016). The MDD was highest in Sughd (88.6%), followed by Dushanbe (87.2%), GBAO (84.2%), DRS (74.6%), and Khatlon (68.5%). There is a pattern of significantly higher food diversity among women living in urban areas (MDD urban: 86.7% vs MDD rural: 77.5%, *p* < .001; Figure 2).

The average duration of exclusive breastfeeding was 5.1 months. Mothers stopped breastfeeding their children on average at the age of 10.8 months (rural: 11.3 and urban: 10.0). The first weaning food for most children was introduced at 6 months and consisted of cereals, potatoes, dairy products, and to some extent eggs. Overall, 60.0% of the children over 6 months had consumed green or black tea in the previous 24 hr, 45.6% received animal milk, and 27.3% sprinkles, containing 15 essential vitamins and minerals (UNICEF catalogue number S0000225). Sprinkles were distributed to around 20 districts from 2012 onwards. In the past 6 months, vitamin A was reported to be received by 78.9% and vitamin D by 31.5% of the 6‐ to 59‐month‐old children.

From 1,523 salt samples, 74.0% were fortified with potassium iodate, 81.0% from urban, and 72.8% from rural households. The highest percentage of households using iodised salt was found is Sughd (88.9%) followed by Dushanbe (82.0%), GBAO (74.5%), DRS (65.7%), and Khatlon (64.3%). In 2003 and 2009, the percentage of households using iodised salt was 52.3% and 84.1%, respectively.

### 3.2 Nutritional status of women 15–49 years

**Figure 3:**
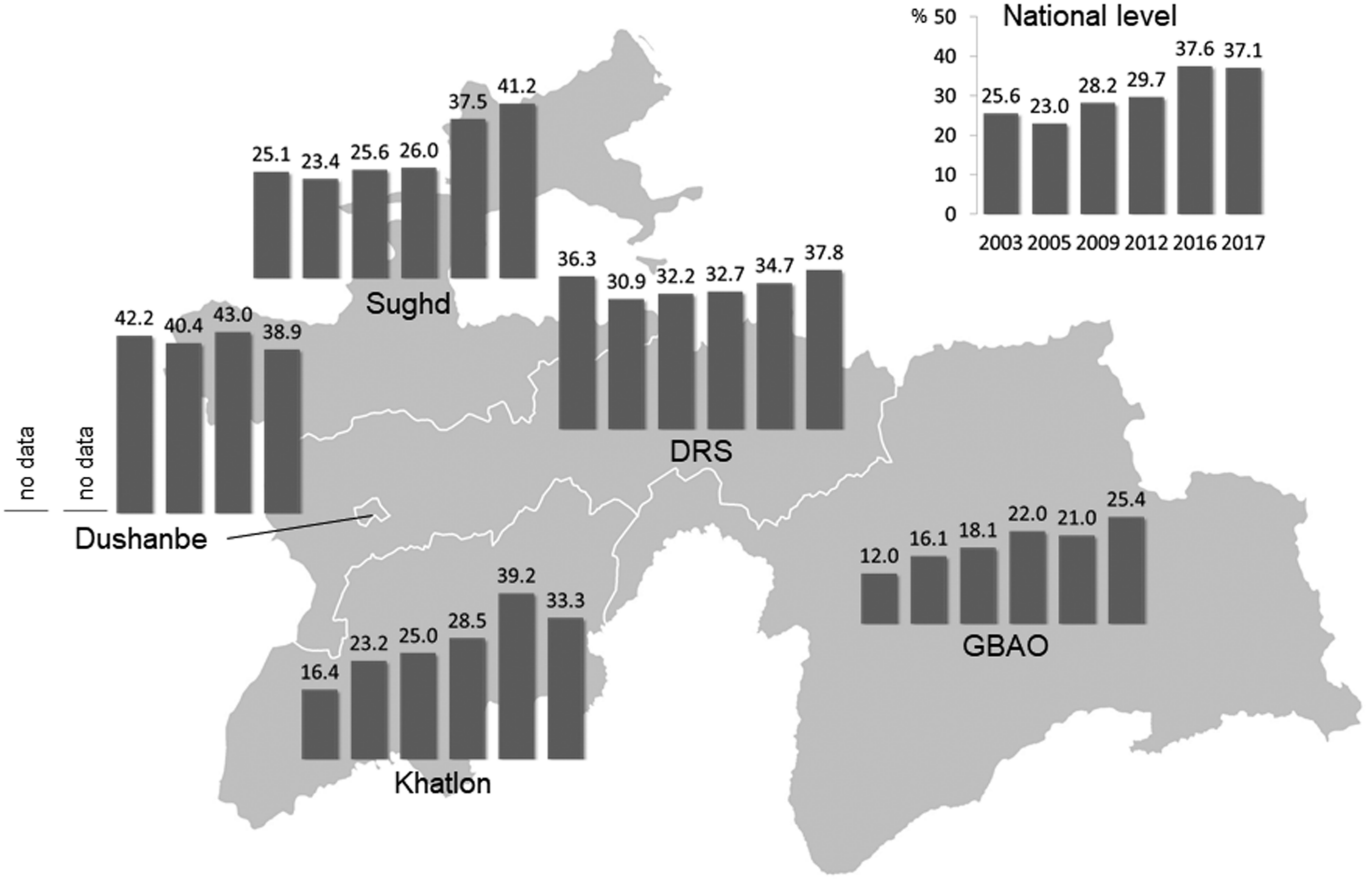
Prevalence of overweight and obesity (body mass index > 25.0 kg/m^2^) from 2003 to 2017 in women 15–49 years

Overall, 8.0% of the women were underweight (BMI < 18.5 kg/m^2^), 24.5% overweight (BMI ≥ 25 to >30 kg/m^2^), and 13.0% obese (BMI ≥ 30 kg/m^2^). Women living in an urban environment had a significantly higher BMI compared with women from rural areas (24.7 kg/m^2^ compared with 23.8 kg/m^2^, *p* < .001). The national prevalence of overweight and obesity had increased steadily from 25.6% in 2003 to 28.2% in 2009, to 37.6% in 2016, and 37.1% in 2017 (Figure 3). In 2016, as in previous years, the highest prevalence of overweight was detected in Dushanbe (43.0%) and the lowest in GBAO (21.0%).

The national prevalence of ID and anaemia in women was 38.0% and 25.8%, respectively, and varied between the oblasts (Table 1). Women living in rural areas had a significant lower iron status compared with those living in urban areas (ID: 40.2% vs 34.0%, *p* = .006; anaemia: 27.8% vs 21.2%, *p* < .001). This was also the case for the mean Hb levels (12.6 g/dl vs 12.9 g/dl, *p* < .001). Further, the prevalence of anaemia was higher in older women (25–40 years: 27.7%, 41–49 years: 27.6%, 15–24: 20.4%, *p* < .001). About half of the anaemia nationally could be attributed to ID (13.8% of 25.8%). The majority of women (61.5%) had a UIC below the cut‐off for sufficient iodine intake (100 μg/L), with inadequate iodine status being more frequent in urban than rural areas (66.8% vs 58.7%, *p* = .001); 12.9% of the women reported taking iodine supplements in the past 6 months. These women were significantly less iodine deficient (53.1%) compared with those not taking supplements (62.9%, *p* = .002). Overall, 46.5% of the women were vitamin A deficient, and 20.5% had insufficient serum folate levels. The prevalence of inflammation was very high across the population (82.5%).

When comparing food intake of anaemic with nonanaemic women, anaemic women consumed significantly less cereals (wheat, bread, rice, pasta, and biscuits, *p* > .05), vegetables (*p* = .003), fruits (*p* = .015), clear broth (homemade soup of chicken or beef bones or meat, often boiled with locally available vegetables, *p* = .006), and tea or coffee (*p* = .006, though here, difference is very small as the vast majority [99%] consumes tea or coffee) compared with nonanaemic women. Women with sufficient UIC were consuming more frequently fish (24.3% vs 5.3%, *p* = .027), nuts and seeds (42.0% vs 35.9%, *p* = .008), clear broth (61.8% vs 56.9%, *p* = .035), fruit juices (*p* = .012), and less plain water (*p* = .003). The reported consumption of eggs was significantly higher in overweight and obese women (*p* = .004), whereas fat and oil consumption was lower (*p* < .000) when compared with normal weight women.

### 3.3 Nutritional status of children 6–59 months

**Figure 4:**
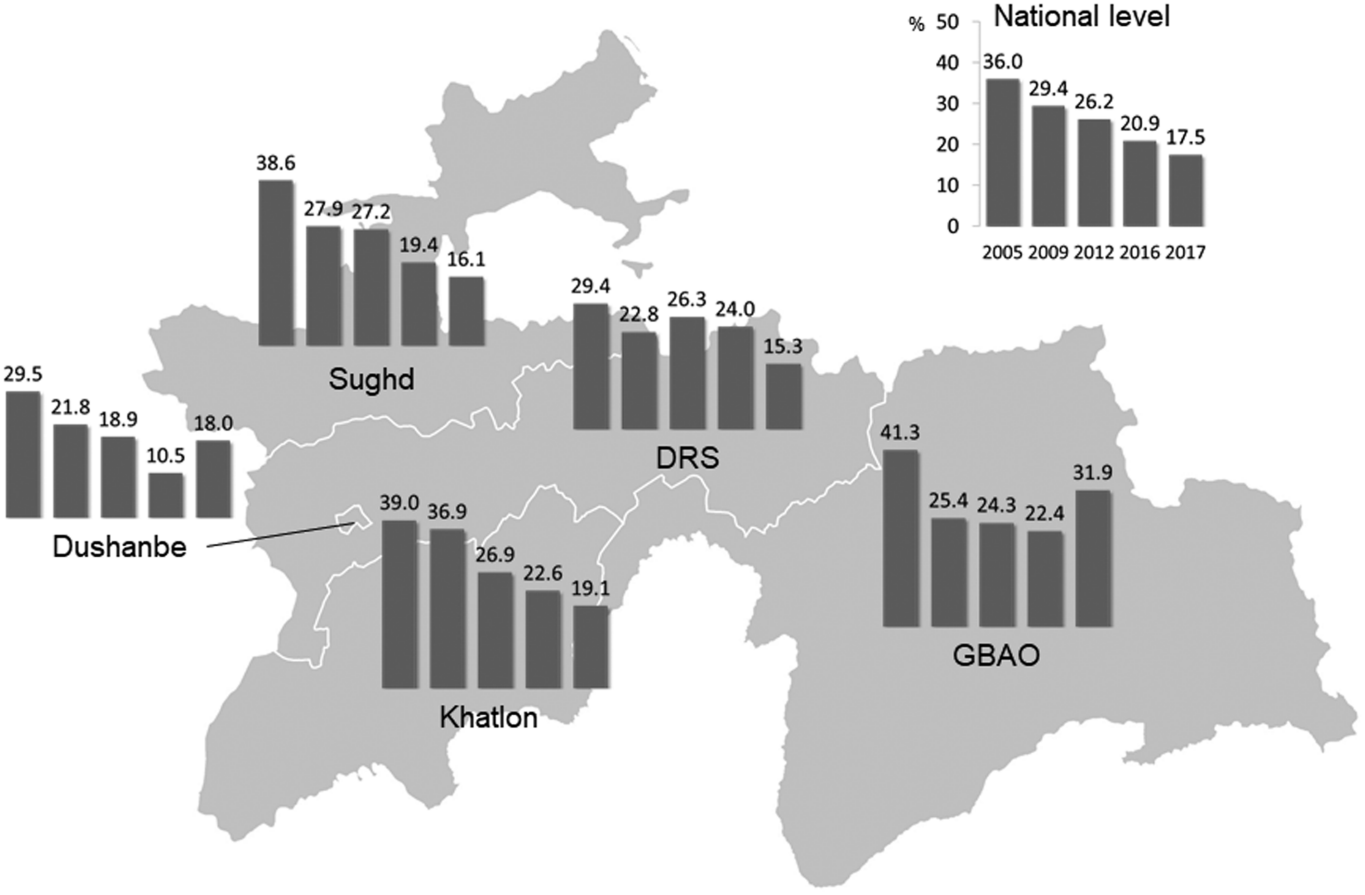
Prevalence of stunting (height‐for‐age z‐score of >−2) in children aged 6–59 months in the years 2003, 2009, 2012, 2016, and 2017

**Figure 5:**
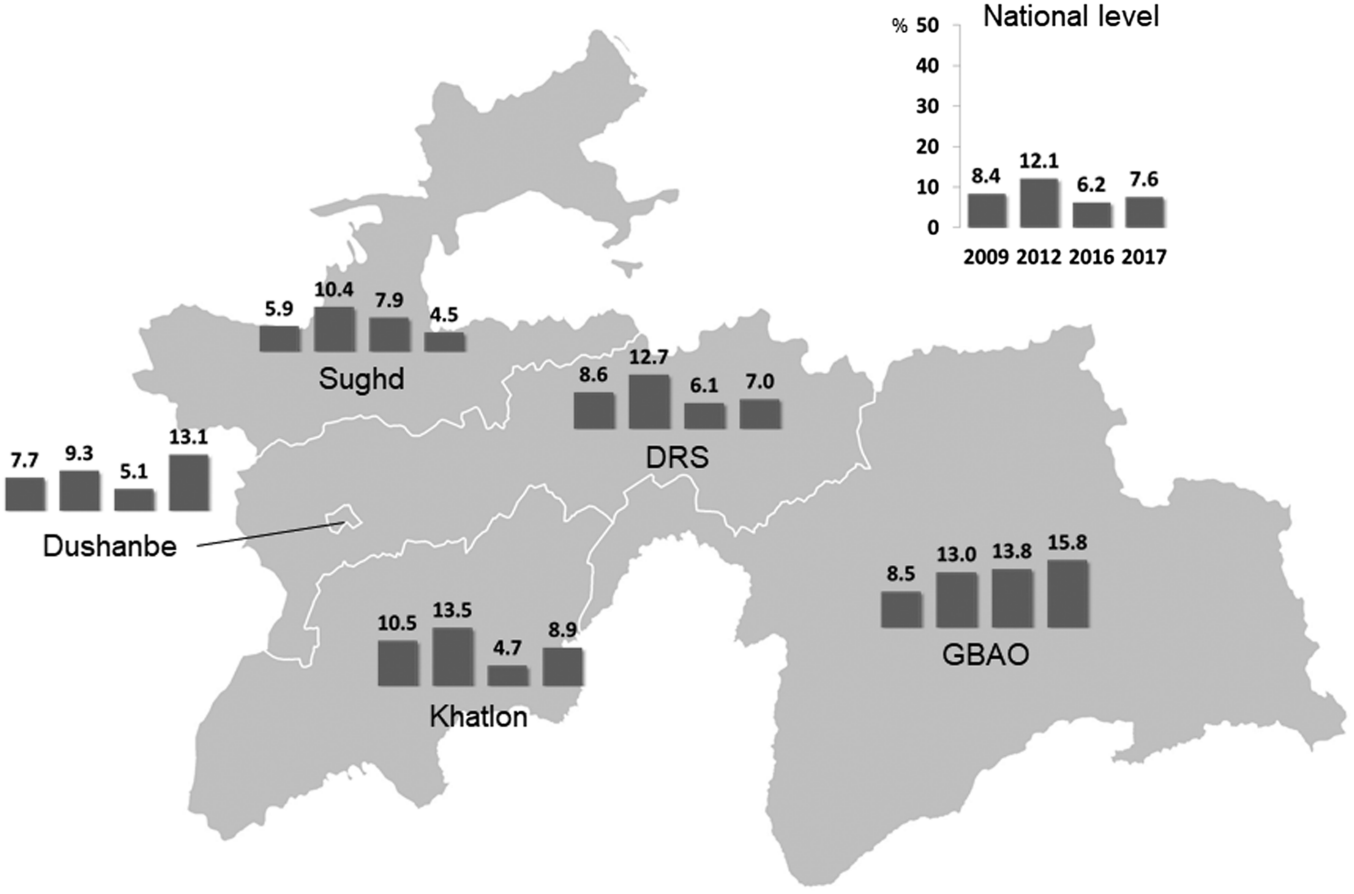
Prevalence of underweight (low weight‐for‐age z‐score of >−2) in children aged 6–59 months from 2009 to 2017

Nationally, 20.9% of the children aged 6–59 months were stunted, 2.8% wasted, and 6.2% underweight. Stunting, wasting, and underweight were significantly higher in the rural population compared with the urban population (23.3% vs 14.2%, *p* < .001; 3.3% vs 1.4%, *p* = .012; 6.9% vs 4.4%, *p* = .035). The national prevalence of stunting is decreasing since 2005, from 36.0% to 17.5% (Figure 4), whereas the prevalence of underweight shows a less clear trend (Figure 5). Interestingly, GBAO and Dushanbe experience an increase in underweight and stunting in recent years.

The national prevalence of ID (52.4%) and anaemia (25.8%) in children varied between the regions and 16.9% of children have IDA (Table 2). ID, anaemia, and IDA are significantly higher in rural areas compared with urban (for all *p* ≤ .001). Half of the children (50.9%) had inadequate UIC, and 37.0% were vitamin A deficient (lowest in Dushanbe 21.0% and highest in Sughd 49.7%, Table 2). Furthermore, 12.4% of children had a vitamin D deficiency. Overall, inflammation was very high (64.0%) across the study population. Supplementation of vitamin A in the past 6 months was more often received by children living in urban compared with rural areas (82.5% vs 77.1%, *p* = .004), although receiving vitamin A supplements was not linked to reduced deficiency. On the other hand, vitamin D supplementation was more often reported from rural children than from urban children (33.2% vs 28.2%, *p* = .021), also here, no significant impact of the supplementation could be seen on deficiency.

### 3.4 Intraindividual double burden

Women's BMI was negatively correlated with ID (*r* = −.067, *p* = .003) and IDA (*r* = −.064, *p* = .005), but positively with inflammation (*r* = .199, *p* < .000). No correlation between the women's BMI and vitamin A, folic acid, or iodine deficiency was found. Underweight women more frequently reported consuming iron supplements compared with overweight women (20.1% vs 13.1%, *p* = .009). For the children, stunting, wasting, and underweight were not correlated with any nutritional deficiency measured.

### 3.5 Household double burden

Women with a low or normal BMI had children living in the same household with lower WHZ (*r* = −.14, *p* = .022), WAZ (*r* = −.13, *p* = .024), lower Hb, and higher rates of anaemia (*r* = −.31 and .89, respectively, both *p* < .001), and overall lower body iron stores (*r* = −1.07, *p* = .038) compared with women with a higher BMI. For example, children living in the same households as women with a mean BMI >25 kg/m^2^ had increased Hb of 0.3 g/dl compared with those living in a household without overweight women. For the other anthropometrical, nutritional and inflammation markers of the children (height‐for‐age, vitamins A and D, SF, TfR, UIC, and CRP), there was no correlation with the BMI of women of the same household.

## 4 DISCUSSION

Overall, our data showed a high prevalence of micronutrient deficiencies, especially iron, iodine, and vitamin A in Tajik women of childbearing age and children aged 6–59 months. One in three women and half of the children were iron‐deficient. Further, two‐third of the women and half of the children had inadequate iodine intake. Also, half of the women and one in three children were vitamin A deficient. These micronutrient deficiencies pose a major public health issue for the women and children of Tajikistan and may impact future work‐force tremendously. More specifically, an anaemia prevalence of 25% of children and women is a moderate public health problem and for the children in GBAO with over 40% being anaemic a severe public health problem. Furthermore, the high prevalence of stunting in children (over 20%) indicates chronic malnutrition already in early life (Black et al., 2008). Although there is a positive trend in the national reduction of stunting from 29.4% in 2009 to 20.9% in 2016 and further to 17.4% in 2017, the current rates remain a public health concern. Furthermore, rising prevalence of overweight and obesity (to 38% in 2016) in Tajik women of reproductive age also have to be recognized as an important public health problem.

In 2016, out of three Tajik women, one was overweight or obese and one was iron deficient. According to our data, this was most probably not the same woman as no intraindividual double burden of malnutrition was identified. A study in Vietnamese women showed that micronutrient deficiencies were observed among all weights (Laillou et al., 2014), whereas in this current study, ID and anaemia were slightly more prominent in underweight women. A study in the Greater Tunis area in Tunisia reported intra‐household double burden of malnutrition, most prominently anaemic children and overweight mothers (Sassi et al., 2018). In the Tajik National Nutrition Survey 2016, there was a strong positive correlation of women's BMI and children's iron status in the same household, and therefore, no evidence of a double burden at household level on this or other assessed indicators.

In Tajik women of reproductive age, BMI was positively correlated with inflammation. This could be due to the so‐called chronic inflammation of obesity, deriving from stimulation of inflammatory mediators through adipose tissue and resulting pro‐inflammatory state and oxidative stress, and finally an increase in CRP (Ellulu, Patimah, Khaza'ai, Rahmat, & Abed, 2017). Inflammation has been shown to decrease ferroportin, through increased production of hepcidin, and therefore reduce iron absorption, as well as the release of storage iron (Nemeth et al., 2004). This can lead to ID and finally to anaemia of inflammation (Cook, 2005). However, this effect could not (yet) be shown in this population, as the obese women indeed had a tendency for increased CRP levels but rather have a better iron status compared with the women with lower BMI. Furthermore, the better iron status in overweight and obese women could not be attributed to more frequent consumption of iron‐rich foods, such as meat, beans, or green leafy vegetables, but may be the result of an overall more stable food availability throughout the year. Of note, the rate of elevated CRP and therefore inflammation was extremely high in the study population. This was partly described in the field reports by many study participants presenting with obvious infections. On the other hand, capillary blood samples are slightly more prone for haemolysis (Heenan, Lunt, Chan, & Frampton, 2017); although this was not noted during handling of the blood specimen, we would like to mention this limitation and its potential impact on increased CRP values.

The national MDD score was adequate with 80.7% of women consuming at least five out of 10 food groups over 24 hr prior to the interview. This was comparable with another study conducted in spring 2016 in Tajikistan, showing similar values for women (87%) but reporting a serious problem in the diversity of children' diet, with only 42% of children aged 6–24 months meeting the WHO guidelines for MDD (Klassen et al., 2019). Unfortunately, children' dietary diversity data are missing in the current study. We identified a strong rural–urban pattern in the nutritional status and MDD of the study population. The reported diet of rural women was significantly less diverse compared with women living in an urban environment. Looking at the specific micronutrient deficiencies, iron, vitamin A, and folate deficiencies were more prominent in rural areas, but lower UICs and higher prevalence of vitamin D deficiency were apparent for urban dwellings. These differences could be explained by first, the most obvious is the different distribution of wealth in the rural and urban households, the DHS 2017 reported that 90% of all urban households, and only 24% of the rural households are in the two highest wealth quintiles (Statistical Agency under the President of the Republic of Tajikistan et al., 2018). Wealth brings lower food insecurity and the possibility to access qualitatively better and more diverse nutrition (fresh nutrient‐rich food [e.g., meat and green leafy vegetables], fortified food, and supplements). Further, working outside in the sun induces cutaneous production of vitamin D; this could be a reason for higher vitamin D values in rural areas (Jablonski & Chaplin, 2018; Webb & Holick, 1988). Then, the better iodine status in rural settings may be due to the more frequent preparation of home‐made meals using iodised salt. A study in China reported people in urban areas preferred noniodised salt in recent years (Zou et al., 2014). This could not be underlined by the current study; In Tajikistan, 99.6% of the urban and 98.7% of rural women knew of the importance of iodised salt in the prevention of goitre (this study, data not shown). Knowledge of other advantages of iodised salt (foetal and mental development) differed a lot between the different oblasts (this study, data not shown). These differences may be due to different information campaigns in the oblasts. About 13% of women reported taking iodine supplements, which was moderately successful to reduce iodine deficiency (from 60% to 50%). Overall, the inadequate iodine intake and the decrease in the percentage of iodised salt in the households over the last years (2009: 84% and 2016: 74%), call for a renewal of the political commitment toward Universal Salt Iodisation, particularly through addressing the persistent problems on the supply side, namely, iodised salt production and quality control and regulation.

Breastfeeding has been reported to reduce the risk of a child–mother pair being undernourished and obese (Oddo et al., 2012). The average time of breastfeeding was slightly longer in rural Tajikistan (11 months) but did not differ statistically from the urban population (10 months).

The high prevalence of micronutrient deficiencies (iron, iodine, and vitamin A) among women and children indicates the continued need for specific public health actions in Tajikistan. Continued emphasis needs to be given to multiple approaches, such as promotion of improved infant and young child feeding practices, flour fortification and in‐home fortification with micronutrient powders (sprinkles), or micronutrient supplementation for children and pregnant women. In line with interventions supported over past years by the Ministry of Health and Social Protection, UNICEF, WHO, WFP, USAID, and other agencies, continued attention to women's and children's nutritional status is required through adopted, cost‐effective, and equitable interventions. Which entail micronutrient supplementation (especially iron, folic acid, and vitamin A), promotion of breastfeeding and age‐specific complementary feeding, salt iodisation, management of severe and acute malnutrition, and design and development a of communication strategy on the first 1,000 days. In September 2013, Tajikistan joined the Scaling Up Nutrition movement. UNICEF together with USAID, as donor coconveners, has been assisting the government in setting up a multisectoral/stakeholder platform as a basis for joint collaborative work towards improving both, nutrition‐specific and nutrition‐sensitive interventions. In the framework of the Scaling Up Nutrition initiative, Ministry of Health and Social Protection with support from international partners, developed a Common Results Framework and a costed multi‐sectoral plan for nutrition.

Overall, the double burden of malnutrition not only adds a layer of complexity to tackle in a population but also gives the opportunity for actions to simultaneously address obesity and undernutrition. For example, promoting breastfeeding and reducing the infant's intake of calories without micronutrients as with breastmilk substitutes and processed complementary foods, can counteract obesity in women and children, as well as chidren's undernutrition and stunting at the same time (Dietz, 2017). Further, emphasis should be given to multidisciplinary collaborative programmes across different sectors, namely, agriculture, education, trade and economy, and health (including WASH). Differences in the rural and urban population seem to be contributing to the national double burden of malnutrition. This calls for an adapted focus on the improvement of infant and child nutrition especially in rural areas but not excluding the urban population. Furthermore, there is a need for a country‐wide information and education campaign to promote a healthy lifestyle, in order to tackle the most important nutritional issues for children and women in Tajikistan.

## CONFLICTS OF INTEREST

The authors declare that they have no conflicts of interest.

## CONTRIBUTIONS

TBJ, LZ, MB, SK, SR, and KW designed research (project conception, development of overall research plan, and study oversight); LZ and SK conducted research (hands‐on conduct of the experiments and data collection); TBJ and KW analysed data or performed statistical analysis; TBJ and KW wrote paper (only authors who made a major contribution); all others have reviewed the final content.
